# Improved uniaxial dielectric properties in aligned diisopropylammonium bromide (DIPAB) doped poly(vinylidene difluoride) (PVDF) nanofibers†

**DOI:** 10.1039/c9ra06470b

**Published:** 2019-10-02

**Authors:** Vaibhav Singh Bhugra, Mohsen Maddah, Grant V. Williams, Natalie Plank, Thomas Nann

**Affiliations:** School of Chemical and Physical Sciences, Victoria University of Wellington Kelburn Parade Wellington 6012 New Zealand; School of Mathematical and Physical Sciences, University of Newcastle University Drive, Callaghan NSW 2308 Australia thomas.nann@newcastle.edu.au

## Abstract

Diisopropylammonium bromide (DIPAB) doped poly(vinylidene difluoride) (PVDF) nanofibers (5, 10 and 24 wt% DIPAB doping) with improved and tunable dielectric properties were synthesised *via* electrospinning. DIPAB nanoparticles were grown *in situ* during the nanofiber formation. X-Ray diffraction (XRD) patterns and Fourier transform infrared spectroscopy (FTIR) proved that electrospinning of DIPAB doped PVDF solutions led to the formation of a highly electro-active β-phase in the nanofibers. Electrospinning in the presence of DIPAB inside PVDF led to very well aligned nanofibers with preferred (001) orientation that further enhanced the effective dipole moments in the nanofiber structures. The dielectric properties of the composite nanofibers were significantly enhanced due to the improved orientation, ionic and interfacial polarisation upon the applied electrospinning process, ionic nature of DIPAB and the interface between the PVDF nanofibers and equally dispersed DIPAB nanoparticles inside them, respectively. The relative dielectric constant of the PVDF nanofibers was improved from 8.5 to 102.5 when nanofibers were doped with 5% of DIPAB. Incorporating DIPAB in PVDF nanofibers has been shown to be an effective way to improve the structural and dielectric properties of PVDF.

## Abstract 2

Diisopropylammonium bromide (DIPAB) doped poly(vinylidene difluoride) (PVDF) nanofibers (5, 10 and 24 wt% DIPAB doping) with improved and tunable dielectric properties were synthesised *via* electrospinning.

## Graphical abstract

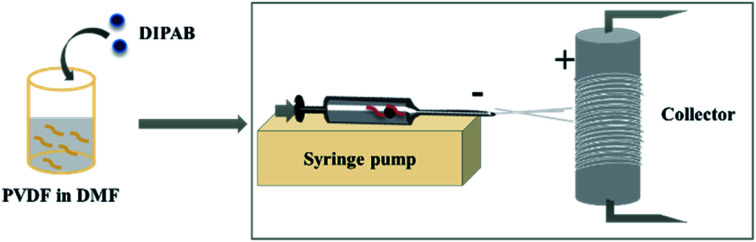

## Introduction

Ferroelectric materials are used in many electronic applications and have been studied for over a century for their multifunctional electro-active properties.^1^ They show spontaneous electric polarisation that can be controlled by an external electric field.^2–4^ Their temperature dependent and electromechanical properties can be used in pyroelectric^5^ and piezoelectric^6^ applications. In recent years, many researchers have started to study organic and molecular ferroelectric materials that became interesting because of their ability to be processed in solutions,^7^ as well as their stability and ease of use.^8^

Poly(vinylidene difluoride) (PVDF) has been a material of interest because it shows distinctive piezoelectric, pyroelectric and ferroelectric properties.^9,10^ It is a semicrystalline polymer that is flexible, lightweight, inexpensive and has a high electrical breakdown strength.^11^ PVDF belongs to the ‘order–disorder’ ferroelectric class in which the phase transition can take place from randomly oriented dipoles (paraelectric phase) to ordered dipoles (ferroelectric phase). PVDF has a crystallinity of around 50% and can be found in five different polymorphs. The monoclinic α-phase of PVDF is the most commonly occurring polymorph with TGTĞ {*Trans*–*Gauche*(+)–*Trans*–*Gauche*(−)} chain conformation. The β-phase shows piezoelectricity with an all-*trans* (TTTT) conformation forming an orthorhombic structure with all the fluorine atoms located on the same side in the polymer crystal structure.^9^ The other polymorphs include γ (orthorhombic; TTTGTTTĞ conformation), δ and ε, which are polar and anti-polar equivalents to α-phase and γ-phase, respectively.

PVDF has a long range of ordered dipole interactions, a strong ferroelectric phase and a Curie temperature, *T*_c_, higher than its melting temperature, *T*_m_.^12^ These properties can be used in a wide range of applications including sensors,^13^ actuators,^14^ capacitors^15^ and non-volatile ferroelectric memories.^16^ The most common α-phase can also be converted easily into β-phase by electrical poling and mechanical drawing.^17–19^ Despite PVDF's potential as an alternative to the inorganic ferroelectric crystals, it suffers from low dielectric constant (∼8.5 for bulk PVDF) resulting in a low electrical energy density that can be stored within the material.^20^ This can be overcome by incorporating materials with high dielectric permittivity such as BaTiO_3_, Ba_*x*_Sr_1−*x*_TiO_3_, Pb(Zr,Ti)O_3_.^21,22^ Although these materials have advantages, they are still heavy metals with a high processing temperature.^23^

Diisopropylammonium bromide (DIPAB) is a molecular, organic and ferroelectric crystal having a room temperature polar point group and a high melting temperature. It exists in two different polymorphs, *i.e.* 1-F and 1-P.^24^ The 1-F (ferroelectric phase) belongs to the monoclinic crystal system, while the 1-P (paraelectric phase) belongs to the rhombohedral crystal system at room temperature. The 1-P phase can be easily converted into 1-F phase by heating DIPAB to 428 K. A spontaneous polarisation of 23 μC cm^−2^ (comparable to that of BaTiO_3_), a high *T*_c_ of 426 K, along with a room temperature dielectric constant of 85 and small loss tangent of 0.44% at the frequency of 400 Hz was reported for pure DIPAB crystals.^23,25–27^ Hence, DIPAB exhibits a good ferroelectric and piezoelectric response along with well-defined ferroelectric domains and can act as a molecular alternative to perovskite ferroelectrics.

Very few PVDF–DIPAB nanocomposites have been reported in the literature and they were all in the form thin films.^9,28^ These publications have demonstrated the nanocomposites' potential portraying an enhanced dielectric strength and a good chemical stability in harsh environments. Electrospinning of the PVDF at low temperatures along with fast solvent evaporation result to β-phase formation and it also influences the crystallinity of the nanofibers. Baji *et al.* showed that electrospinning is said to enhance ferroelectric and piezoelectric phase of PVDF because of uniaxial stretching taking place at high electric potentials.^29^ The diameter, surface tension and mechanical strength of the fibers are impacted by the electrospinning parameters and type of solvent used. So far, DIPAB–PVDF composites as nanofibers have not been reported.

We studied the properties of uniaxially aligned DIPAB doped PVDF nanofibers formed using rotating drum electrospinning. DIPAB was used along with the PVDF due to its high dielectric constant value and affinity to form *in situ* nanocrystals with high surface area. The high β-phase PVDF nanofibers with varying loading content of DIPAB have been synthesised and studies with respect to the dielectric properties of the formed structures have been investigated. We found out that there was an improvement in the relative dielectric constant of the DIPAB doped PVDF nanofibers by more than 12 times in comparison to the regular bulk PVDF and this value is higher than the reported value of DIPAB–PVDF films reported earlier in the literature.^9,28^

## Experimental

### Materials

All chemicals that were used in the reaction were of analytical grade. The poly(vinylidene difluoride) (HSV 900) in powder form was purchased from MTI chemicals, USA. *N*,*N*-dimethylformamide (DMF), diisopropylamine and hydrobromic acid (HBr) were purchased from Sigma Aldrich.

### Synthetic procedures

#### Synthesis of diisopropylammonium bromide

Diisopropylammonium bromide (DIPAB) crystals were obtained by the slow evaporation of the equimolar solution of diisopropylamine and hydrobromic acid in methanol. The obtained DIPAB crystals were collected and finely ground using mortar and pestle, and stored under inert gas atmosphere.

#### Preparation of DIPAB doped PVDF solutions for electrospinning

A 9.8 wt% homogenous solution of PVDF powder in DMF was obtained by heating the mixture at 105 °C for 1.5 hours. Different amounts of DIPAB were then added to the solution. The mixtures were left to stir at room temperature overnight to ensure complete dissolution of DIPAB. The overnight stirring also leads to the removal of air bubbles in the solution.

**Fig. 1:**
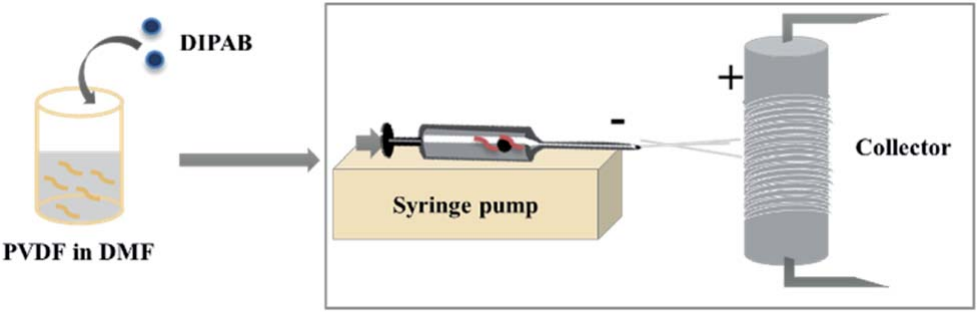
Electrospinning setup for DIPAB–PVDF nanofibers.

DIPAB doped PVDF solution (10 ml) with a doping ratio 0%, 5%, 10% and 24% by wt were prepared. The following doping ratios were considered based on the previous studies performed on DIPAB doped PVDF thin film samples where the highest dielectric constant was observed in samples below 10 wt% DIPAB doping.^9^ The sample with highest DIPAB loading (24.2 wt%) was considered based on the improved orientation in doping samples since the above this concentration, the polymer solution became too viscous to electrospin well and it was difficult to maintain the fibers orientation. Each sample was loaded in a plastic syringe fitted with a metallic needle tip (18G). The syringe containing the solution was then loaded on the top of an automatic syringe pump (Harvard Apparatus) and was connected to the high voltage power source (Gamma High Voltage Research) as shown in Fig. 1. The solution was fed to the setup at varying infusion rates and electric fields depending on the DIPAB–PVDF ratio (Table 1). The fibers were collected on an aluminum foil wrapped on a custom made rotating drum (diameter – 7 cm) located at a fixed distance of 7 cm.

**Table 1:** Parameters for the electrospinning of PVDF nanofibers with varying DIPAB content by wt%

S. no.	Sample label	DIPAB in PVDF [wt%]	Infusion rate [ml h^−1^]	Voltage applied [kV]
1	DIPAB 0%	0%	1	12
2	DIPAB 5%	5%	0.85	13
3	DIPAB 10%	10%	0.75	10.7
4	DIPAB 24%	24%	0.85	15

### Characterization

X-Ray diffraction (XRD) was performed on a Panalytical X-ray diffractometer using Cu-Kα at an operating voltage of 45 kV and current of 40 mA at room temperature to study the crystal structure of the nanocomposite fibers. Fourier transform infrared spectra (FTIR) was recorded over a range of 650 to 1000 cm^−1^ to study the presence of β-phase of PVDF. Scanning electron microscopy (SEM) was performed on a Jeol SEM-6500 microscope to study the morphologies of obtained nanofibers. The samples were coated with Platinum to prevent charging. The XRD, FTIR and SEM were performed on the sheets of aligned PVDF nanofibers with different DIPAB ratios. The dielectric measurements were performed on the same batch of the samples. High resolution transmission electron microscopy (HR-TEM) was further performed using a Jeol TEM-2100F instrument to confirm the uniform doping of DIPAB and observe the formation of DIPAB nanocrystals inside the fibers. The composite nanofibers were spun directly on carbon coated lacey copper grids. The uniform doping of DIPAB was also proven by performing an Energy dispersive X-ray spectroscopic (EDXS) analysis on the sample using the same equipment for SEM imaging. The dielectric properties of the nanofibers were measured using parallel RLC circuit with Agilent 4294A impedance analyser and Agilent 16451B dielectric test fixture at 500 mV for all the measurements. The impedances of nanofibers were measured in the range of 40 Hz to 1 MHz. Smoothening was performed on the collected dielectric data to reduce the noise. The observed noise might have been caused by the improper contact of the electrodes with some nanofibers during the measurements.

## Results and discussions

### Structural characterization

**Fig. 2:**
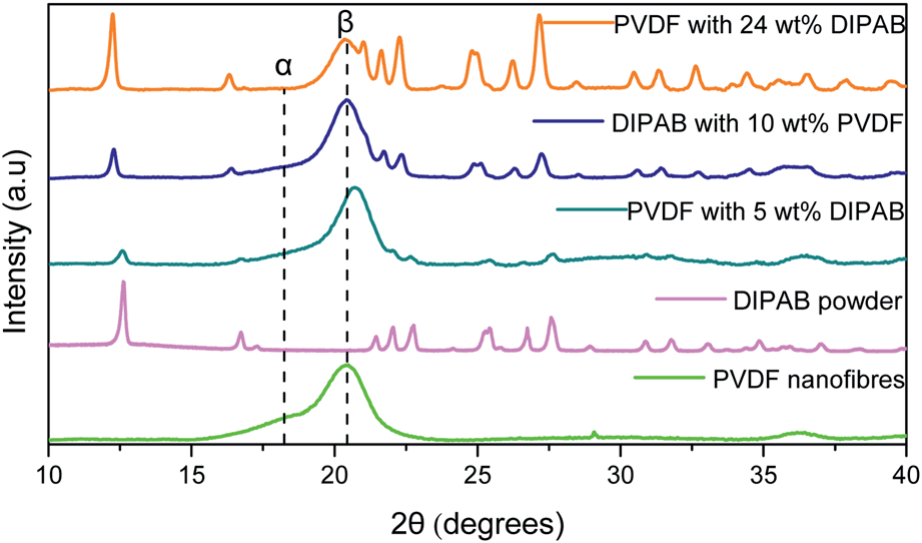
XRD patterns of PVDF nanofibers, DIPAB and DIPAB–PVDF nanofibers showing the dominating β-phase in DIPAB–PVDF nanofibers sample.

Fig. 2 shows the comparison of X-ray diffraction (XRD) patterns of PVDF nanofibers, DIPAB powder, and DIPAB–PVDF nanofibers. The XRD of the PVDF nanofibers shows a strong diffraction peak at 20.3°, which corresponds to the β-phase of PVDF. No significant non-polar α-phase peak at 18.3° and γ-phase peak at 38.3° of PVDF were observed in the nanofiber samples containing DIPAB. A small peak was observed at 18.3° for the PVDF sample without DIPAB. The peak of the β-phase shifts slightly to 20.42° when it is loaded with DIPAB powder probably because of the interaction of DIPAB with the PVDF structure. These results also confirm the DIPAB incorporation into the PVDF matrix. The α-phase or the 1-F (ferroelectric) phase of DIPAB is clearly visible through the presence of characteristic 2*θ* peaks in the XRD at 12.5°, 17.0°, 22.4°, 25.2° and 27.6°. These peaks correspond to the monoclinic system of DIPAB crystals.

**Fig. 3:**
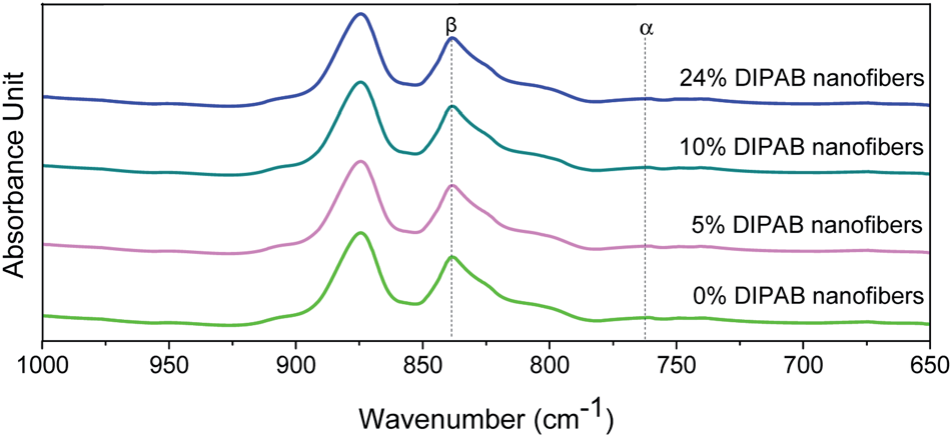
FTIR spectra of DIPAB–PVDF composite films for varying weight ratios 0–24% showing the formation of the dominating electroactive β-phase in electrospun nanofibers.

The crystalline structure and β-phase of PVDF nanofibers with different concentrations of DIPAB was investigated by Fourier transform infrared (FTIR) spectroscopy. Fig. 3 shows the FTIR spectra for all the nanofiber structures. The vibration bands at 510 cm^−1^ (CF_2_ stretching), 598 cm^−1^ (CF_2_ waging) and 840 cm^−1^, (CF_2_ asymmetric stretching and CH_2_ rocking) represent the electroactive β-phase. The electrospinning of nanofibers result to higher β-phase content when it is performed at room temperature (below 80 °C) along with uniaxial stretching at high electric fields. The intense vibration occurring at 840 cm^−1^ confirms the presence of a very high concentration of the β-phase. The β-phase was calculated for all the nanofibers sample using the equation:
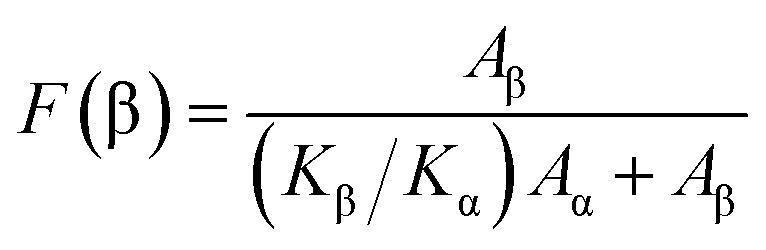
where *A*_α_ and *A*_β_ are the corresponding wavenumber values at 762 cm^−1^ and 840 cm^−1^, and the *K*_α_ and *K*_β_ are the absorption coefficients for the α and β-phases with a value of 6.1 × 10^4^ cm^2^ mol^−1^ and 7.7 × 10^4^ cm^2^ mol^−1^, respectively.^30^ The high β-phase of ∼93.8 wt% was observed in all the DIPAB doped PVDF samples.

**Fig. 4:**
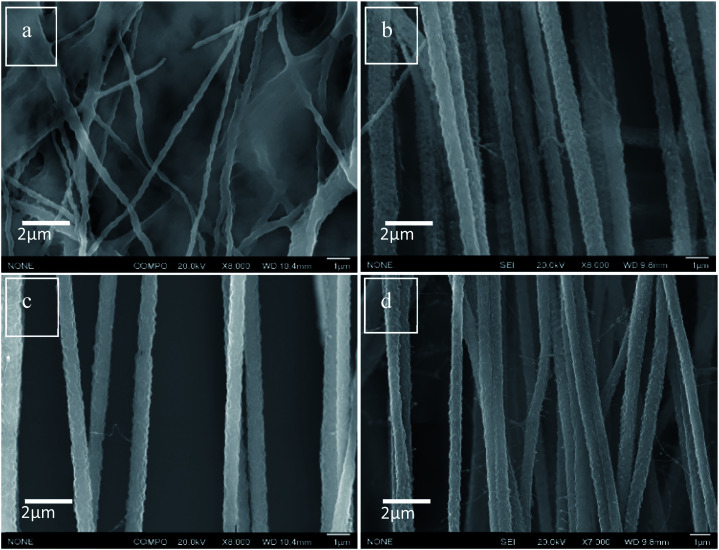
SEM micrographs of (a) DIPAB 0%, (b) DIPAB 5%, (c) DIPAB 10% and (d) DIPAB 24% representing alignment in PVDF nanofiber samples doped with DIPAB.

The incorporation and dispersion of DIPAB crystals inside the PVDF nanofibers was confirmed using Scanning Electron Microscopy (SEM). Fig. 4(a) shows the SEM micrograph of nanofibers with pure PVDF. The alignment and size distribution of pure PVDF nanofibers were not uniform. Fig. 4(b–d) show that the nanofibers were aligned when 5 wt%, 10 wt% and 24 wt% DIPAB doping was applied. At higher DIPAB concentrations in the PVDF solutions, the fibers' alignment degraded. The alignment issues were corrected by adjusting the infusion rate of polymer solutions and the applied voltage. The studies were performed on four electrospun samples based on the wt% of DIPAB added to the PVDF solution in DMF. The maximum doping of DIPAB in PVDF prior to electrospinning was kept around 24.2 wt% after which it became very difficult to obtain well aligned nanofibers. DIPAB was found to be everywhere in the fibers and not in the background (see ESI†). The signal of nitrogen and bromine confirmed the presence of DIPAB in the nanofibers.

**Fig. 5:**
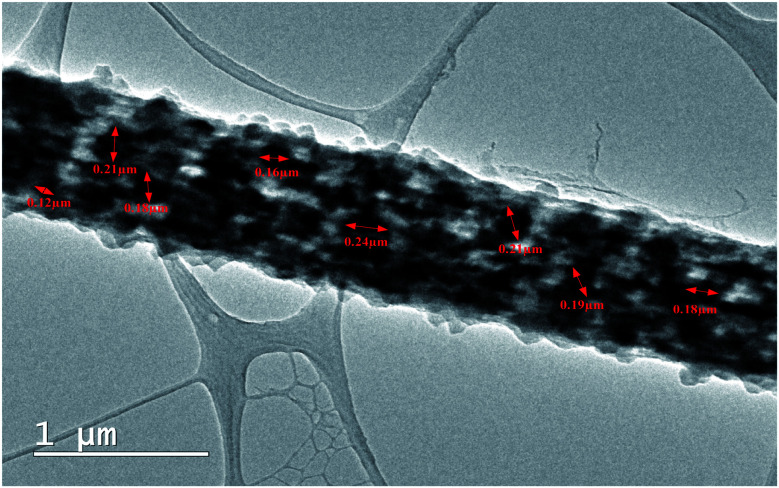
TEM micrograph of single DIPAB–PVDF (DIPAB 5%) nanofiber incorporated with nanoparticles of DIPAB.

High resolution Transmission Electron Microscopy (TEM) was further performed to confirm the uniform doping of DIPAB in the PVDF nanofibers. Fig. 5 shows the TEM micrograph of a single DIPAB–PVDF nanofiber containing 5 wt% DIPAB. The darker contrast material is DIPAB while lighter contrast is portrayed by semicrystalline PVDF. DIPAB was found to be dispersed equally inside the nanofibers, which creates a large interface between the two materials without any visible agglomeration. The size of the DIPAB nanoparticles formed *in situ* was in the range of ∼80–250 nm.

### Dielectric and ferroelectric properties

**Fig. 6:**
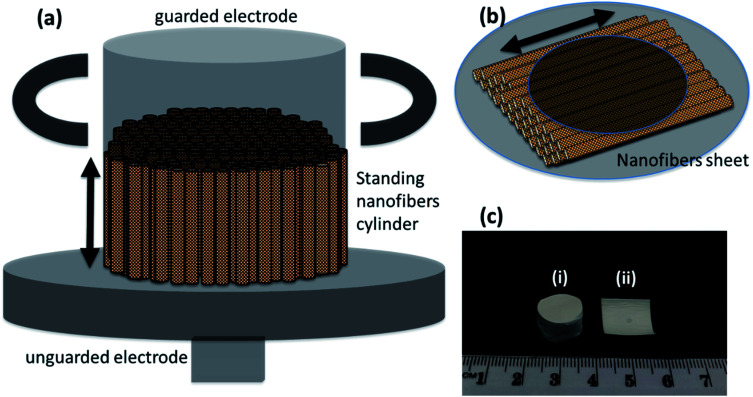
Schematic for electrical measurements, (a) cylinder of standing electrospunned nanofibers in between dielectric test fixture (Agilent 16451B) electrodes for dielectric measurements parallel to the length, (b) sheet of nanofibers for dielectric measurements perpendicular to the length, and (c) real life sample for dielectric measurements (i) nanofibers cylinder (ii) nanofibers sheet.

The frequency dependent measurements were performed parallel as well as perpendicular to the nanofibers' orientation. This was done to prove the enhanced performance of nanofibers in the preferred orientation. The measurements parallel to the nanofibers required the formation of a cylinder of standing nanofibers with a height of less than 1 cm—Fig. 6(a). The cylinder of standing nanofibers was formed by stacking multiple sheets of parallel nanofibers on the top of each other. These sheets were obtained by neatly cutting the big electrospunned nanofibers sheet into smaller sheets. The characterisation and measurements were also performed on the same sample. The utmost care was taken that the orientation was preserved in the overall cylinder. The nanofiber sheets were held together and packed carefully from the side using Teflon tape. The diameter of the cylinder of standing nanofibers was bigger than that of the electrode. Therefore, the Teflon tape had no impact during the measurement of the dielectric properties of DIPAB doped PVDF nanofibers. The volume fraction measurements were later performed individually on each sample to provide accurate results. The measurements perpendicular to the nanofibers' orientation were done by placing the nanofiber sheets between the two electrodes of the Agilent 16451B dielectric test fixture—Fig. 6(b). Fig. 6(c) shows a nanofiber cylinder and sheet made by the above method. Care was taken that the nanofiber cylinders retained their shape and alignment during the measurements. This method was chosen to provide more accuracy in the data by using the same electrodes and setup for all the frequency dependent measurements. Since all of the samples were spun at different infusion rates, we expected them to have different thicknesses when in sheet form. The height of the cylinders was also carefully measured. Table 2 shows the thicknesses and heights of the film samples and cylinder samples, respectively.

**Table 2:** Representation of the thickness (*d*) in mm for DIPAB–PVDF nanofibers samples for dielectric measurements

S. no.	Sample name	Thickness (*d*) [mm]
1	DIPAB 5% film	0.17
2	DIPAB 10% film	0.12
3	DIPAB 24% film	0.20
4	DIPAB 5% cylinder	1.30
5	DIPAB 10% cylinder	1.22
6	DIPAB 24% cylinder	6.30

The capacitance (*C*), loss tangent (tan *δ*), admittance magnitude (*Y*) and phase (*θ*) of the samples were measured to calculate the relative dielectric constant (*ε*_r_) and the AC conductivity (*σ*_AC_) using the following equations:

1 *ε*_r_ = *Cd*/(*ε*_0_*A*)

2 *σ*_AC_ = *Yd* cos(*θ*)/*A*

where, *d* and *A* represent the thickness and area of the samples, respectively, and *ε*_0_ is the constant vacuum permittivity with a value of 8.854 × 10^−12^ F m^−1^.

The dielectric values were calculated based on the volume fraction of electrospun nanofibers as the nanofiber sheets had voids in them. Eqn (10) was used to calculate the volume fraction of nanofibers during the measurements and the actual relative dielectric constant was measured using eqn (11). These equations were derived using eqn (3)–(9).

3 *m*_f_ = *m*_p_ + *m*_d_

4 *m*_p_ = *x*(*m*_s_)

5 *m*_d_ = 1 − *x*(*m*_s_)

6
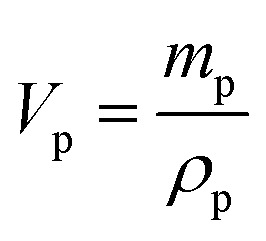
7
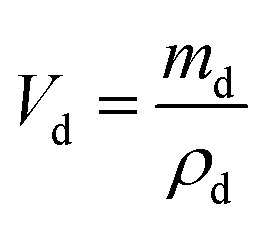

8 *V*_f_ = *V*_p_ + *V*_d_

9 *V*_s_ = π*r*_s_^2^*d*

10
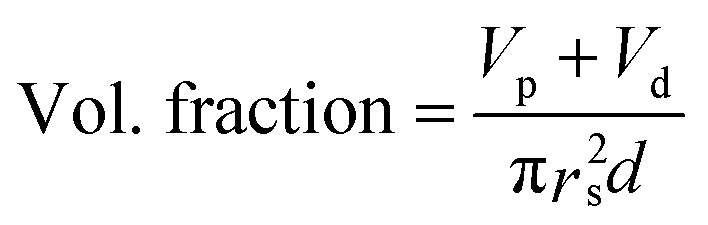
11
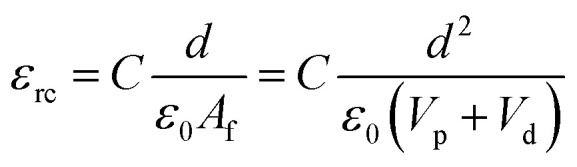
*m*_f_, *m*_p_ and *m*_d_ are the masses, and *V*_f_, *V*_p_ and *V*_d_ are the volumes of electrospun nanofibers, PVDF and DIPAB, respectively. *V*_s_ is the overall volume of the samples. *ε*_rc_ is the corrected relative dielectric constant of the sample (eqn (11)).

**Fig. 7:**
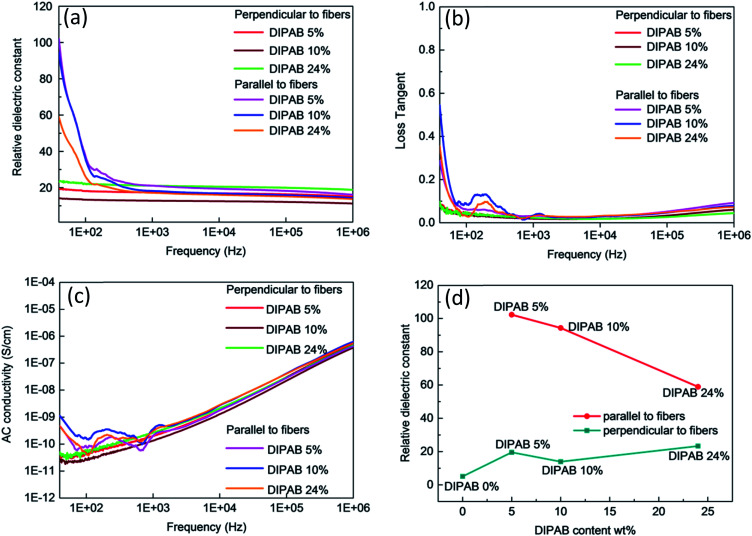
Frequency dependent dielectric properties of DIPAB–PVDF nanofibers: (a) dielectric constant, (b) loss tangent (c) AC conductivity, (d) DIPAB content dependence of dielectric constant of DIPAB–PVDF nanofibers.

The frequency dependent properties of DIPAB doped PVDF nanofibers with varying DIPAB content ratio at room temperature in the range of 40 Hz to 1 MHz is shown in Fig. 7. DIPAB doped PVDF nanofibers showed an enhanced dielectric constant in comparison to the previously reported dielectric constants for pure PVDF nanofibers which were in the range of 11 to 18 at low frequencies.^31–33^Fig. 7(a) shows the variations in the dielectric constant of PVDF nanofibers doped with varying ratios of DIPAB. The data shows a decrease in dielectric constant with increasing frequency in all of the nanofiber samples due to the decreasing effective number of dipoles.^34,35^ The dipoles' oscillations at lower frequencies are in sync with the alternating field, while at higher frequencies they are not. This results in a reduction in the effective number of dipoles. The DIPAB nanocrystals dispersion inside the PVDF nanofibers results to large interfacial area between the two materials. Due to the large difference in the electrical conductivities of PVDF and DIPAB, the charges can migrate and accumulate at the interface of the materials on the application of the electric field. This results in interfacial polarisation, and hence a relatively high dielectric constant.^36^ With increasing DIPAB content, the inter-particle distance decreases and the interfacial area per unit mass increases.^37^ This results in the improvement of the average polarisation associated with the coupling between neighboring grains leading to a significantly higher dielectric enhancement of the composite nanofibers. However, this is true only at low concentrations of DIPAB doping (<5 wt%). The interfacial polarisation works smoothly at lower frequencies as the charges have enough time to move. At higher frequencies, the mismatch between frequency and charge carrier movements results in a decrease in dielectric constant.^38^ The dielectric constant was also affected positively when DIPAB was added to PVDF because of the highly ionic nature of the DIPAB crystals that contributed to the dielectric constant by ionic polarisation.

The two-way relative dielectric constant measurements (parallel as well as perpendicular to the nanofibers) were undertaken to prove the alignment of dipoles along the direction of the nanofibers. During the electrospinning process, the PVDF nanofibers were spun under a very high applied voltage (ranging from 10 kV to 17.5 kV). This resulted in the poling in the PVDF structure where the alignment of the dipoles is along the direction of spinning of nanofibers that cause an increase in the effective dipole moment in these structures. This phenomenon is known as orientation polarisation. It can be seen from Fig. 5 that addition of DIPAB to the PVDF has resulted in very well aligned nanofibers which increased effective dipole moments along the length or parallel to the nanofibers, and hence higher relative dielectric constants.

The maximum dielectric constant recorded was ∼102 in 5 wt% DIPAB doped PVDF (DIPAB 5%) nanofiber samples when the measurements were performed parallel to the nanofibers. To the best of our knowledge, this was by far the highest value ever recorded for this composite material. The dielectric value when measured perpendicular to the nanofibers was much lower (∼19 at 40 Hz) for the DIPAB 5% sample. This proves that most of the dipoles present in the nanofibers were contributing to the dielectric behavior of nanofibers in the direction along the length of the nanofibers showing the effect of orientation polarisation. The values are lower in all the samples when measured perpendicular to nanofibers and they followed the trend like other nanofibers with increasing frequency. It is expected that dielectric constant should increase with the increase in the doping of DIPAB in the PVDF nanofibers owing to the high spontaneous ionic polarisation of DIPAB. The dielectric constant of DIPAB 5 wt% sample was observed to be the highest at low frequencies as a result of the formation of DIPAB nanoparticles that are equally dispersed in the nanofibers. Fig. 7(a) shows the dielectric constant of the higher DIPAB doped samples (*e.g.* DIPAB 24%) to be lower than DIPAB 5% sample. This can be attributed to the formation of in larger DIPAB particles at higher doping. The increased DIPAB particle size results to decreased surface to volume ratio and hence decreased surface area between the two materials. The decreased surface area resulted to a lower interfacial polarisation as it directly dependent on the interface area. The dielectric constant should increase with the increasing DIPAB concentration since DIPAB has relatively higher *ε*_r_ but the decreasing trend of *ε*_r_ proved that the ionic polarisation was dominated by the interfacial polarisation. This proves domination of interfacial polarisation at low frequencies. The ionic polarisation exceeds the interfacial polarisation at high DIPAB concentration. This can be confirmed by the dielectric measurements performed perpendicular to the alignment of the nanofibers. The dielectric constant was found to be highest in the DIPAB 24% sample. The dielectric measurements performed parallel to the nanofibers proved that interfacial polarisation play a huge role in enhancing the dielectric properties. The ionic and interfacial polarisation can usually be attributed to the distinguishable dielectric loss factor peaks. However, the complex network of DIPAB–PVDF in this work cannot be represented by a simple model and the specific dielectric loss factor peaks associated with different polarisations could not be plotted. The individual fibers and the conjunction between the fibers corresponded to multiple peaks where individual peaks were indistinguishable from each other. Overall, the dielectric constant is most effected by orientation of dipoles followed by the interfacial polarisation as a result of charge trapping followed by the ionic polarisation.

The loss tangent *vs.* frequency values are shown in Fig. 7(b). The loss tangent values decreased with increasing frequency as expected due to the Maxwell–Wagner–Sillars effect.^28^ The DIPAB 5% sample had a loss tangent of 0.3 at 40 Hz parallel to the nanofibers. The highest loss tangent of 0.55 was shown by the DIPAB 10% sample at low frequency. The loss tangent for all the samples measured perpendicular to the nanofibers' orientation was very low (<0.1). The loss tangent tends to increase at higher frequency, which is caused by conductance. The AC conductivity increased with increasing frequency as shown in Fig. 7(c). It was found to be highest in the DIPAB 10% sample with a value of 1.1 × 10^−9^ S cm^−1^ at 40 Hz. The DIPAB 5% sample had an AC conductivity of 0.43 × 10^−9^ S cm^−1^ at 40 Hz. The bump in the dielectric constant, loss tangent and AC conductivity data for all the samples was observed in the range of 100 Hz to 500 Hz when the measurements were performed parallel to the nanofibers. These features are constant in all the sample which proves that it is not the noise. It could be the result of complex pathways formation in the composite structure. The limit of detectability of the impedance analyser, for example, the AC conductivity measurements where the value is as low as 2.2 × 10^−10^ S cm^−1^ for DIPAB 5% sample makes it difficult to conclude the real reason for the above feature. Fig. 7(d) shows the comparison of relative dielectric constants of all the fibers parallel and perpendicular to the nanofibers for varying DIPAB doping in the nanofibers at 40 Hz. The DIPAB 5% sample had the highest relative dielectric constant. The value of *ε*_r_ decreased gradually with an increasing amount of DIPAB doping. The values of *ε*_r_, when measured perpendicular to the nanofibers, were very low for the same fibers because of a very low number of effective dipoles. This also proves that the orientation polarisation and presence of DIPAB play a significant role in the enhancement of the dielectric properties of the material.

## Conclusions

For the first time DIPAB, which is a highly ferroelectric compound was successfully dispersed in PVDF nanofibers. The electrospinning and addition of DIPAB helped to obtain very high β-phase, improved alignment and crystallinity in the fibers in comparison to the thin-film samples. The dielectric properties were significantly improved and by far the highest relative dielectric constant was observed in this composite material. Incorporating DIPAB in PVDF is an effective way to produce anisotropic and highly dielectric nanofibers that may be ideal candidates for applications such as electric-energy-storage capacitors and actuators.

## Conflicts of interest

There are no conflicts to declare.

## Supplementary Material

RA-009-C9RA06470B-s001
