# Supplementary material for: Improved uniaxial dielectric properties in aligned diisopropylammonium bromide (DIPAB) doped poly(vinylidene difluoride) (PVDF) nanofibers
Source: RSC Adv. 2019 Oct 2;9(54):31233–40. doi: 10.1039/c9ra06470b (PMC9072744; doi:10.1039/c9ra06470b)
Supplement: RA-009-C9RA06470B-s001 [file RA-009-C9RA06470B-s001.pdf]

Electronic Supplementary Material (ESI) for RSC Advances.
This journal is © The Royal Society of Chemistry 2019

Improved uniaxial dielectric properties in aligned diisopropylammonium bromide (DIPAB) doped Poly-(vinylidene difluoride) (PVDF) nanofibers

Vaibhav Singh Bhugra,[a] Mohsen Maddah,[a] Grant V. Williams,[a] Natalie Plank,[a] and Thomas Nann*[b]

Supplementary information

The elemental mapping at high magnification using Scanning electron microscopy (SEM) setup with energy dispersive X-Ray spectrometry (EDS) was performed on DIPAB doped PVDF nanofibers sample to study the presence of DIPAB inside the PVDF nanofibers. Figure 1 shows that the elemental signals of Bromine and Nitrogen from DIPAB coincides with the elemental signals observed from PVDF structures (Fluorine).

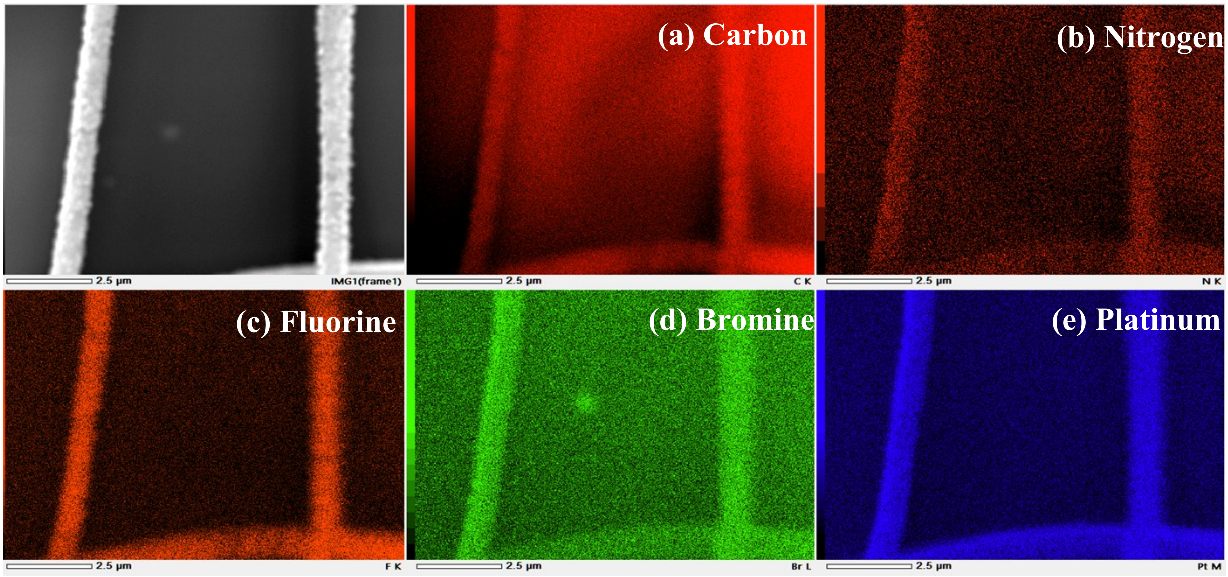

Table 1 - EDXS of DIPAB 5% composite nanofibers sample. The signals from DIPAB having (b) Nitrogen and (d) Bromine coincides with (c) Fluorine from PVDF proving the presence of DIPAB within the nanofibers.
